# Significance of Oral Care for Children with Autism Spectrum Disorder—A Narrative Literature Review

**DOI:** 10.3390/children12060750

**Published:** 2025-06-09

**Authors:** Sirma Angelova, Desislava Konstantinova, Anna Nenova-Nogalcheva, Rouzha Pancheva

**Affiliations:** 1 Department of Pediatric Dentistry, Faculty of Dental Medicine, Medical University “Prof. Dr. Paraskev Stoyanov”, 9000 Varna, Bulgaria; 2 Department of Dental Materials and Prosthetic Dentistry, Faculty of Dental Medicine, Medical University “Prof. Dr. Paraskev Stoyanov”, 9000 Varna, Bulgaria; desi.konstantinova@mu-varna.bg; 3 Department of Oral Surgery, Faculty of Dental Medicine, Medical University “Prof. Dr. Paraskev Stoyanov”, 9000 Varna, Bulgaria; anna.nenova@mu-varna.bg; 4 Department of Hygiene and Epidemiology, Faculty of Public Health, Medical University “Prof. Dr. Paraskev Stoyanov”, 9000 Varna, Bulgaria; ruja.pancheva@mu-varna.bg

**Keywords:** autism spectrum disorder, children, oral care, dental practitioners, parents

## Abstract

Background: Autism spectrum disorder (ASD) is a neurodevelopmental condition in children that typically involves challenges in cognition, behavior, and communication. While many children with ASD exhibit significant impairments in both verbal and non-verbal communication, the severity and nature of these difficulties can vary widely. In addition to its impact on overall health, ASD also affects oral health, leading to increased vulnerability to dental disease. Aim: This narrative review aims to summarize key oral health challenges and care strategies for children with ASD, focusing on clinical risks, behavioral barriers, caregiver roles, and effective interventions. Materials and Methods: A comprehensive literature search was conducted using four databases—PubMed, Scopus, Web of Science, and Google Scholar—as well as relevant study registries where applicable. Peer-reviewed articles published in English between 2010 and 2024 were identified using keywords and their synonyms, such as autism spectrum disorder, children, oral care, dental practitioners, and parents. Studies were included based on relevance to oral health challenges and interventions in children diagnosed with ASD. Results: Children with ASD experience a range of sensory sensitivities, attention deficits, hyperactivity, and behavioral resistance, which significantly hinder the performance of adequate oral hygiene practices. These challenges contribute to a lack of effective dental prophylaxis and limited access to regular preventive care, ultimately resulting in poorer oral health outcomes and reduced oral health-related quality of life. Conclusion: Due to the multifaceted characteristics of ASD, children with this condition face significant barriers in accessing appropriate and individualized oral care. This increases their risks of developing oral health disorders, underscoring the need for coordinated efforts between caregivers and dental professionals to improve oral health outcomes in this vulnerable population.

## 1. Introduction

Autism spectrum disorder (ASD) is a complex neurodevelopmental condition characterized by persistent challenges in social communication and interaction and restricted, repetitive patterns of behavior. Globally, the prevalence of ASD has been increasing, accompanied by growing recognition of the broader health challenges faced by individuals with autism, including oral health complications.

Children with ASD frequently experience significantly poorer oral health outcomes compared to their neurotypical peers. Multiple studies have documented higher rates of dental trauma, untreated caries, gingival inflammation, and oral infections among children on the autism spectrum, largely attributed to inadequate oral hygiene, behavioral difficulties during oral care routines, and sensory sensitivities [1,2]. These children often require specialized dental interventions but face numerous barriers in accessing dental care, such as the limited availability of trained professionals, communication difficulties, and environmental challenges within dental settings [2].

The World Health Organization defines oral health as the state of being free from chronic mouth and facial pain, oral infections and sores, periodontal disease, tooth decay, and other diseases and disorders that limit an individual’s capacity in biting, chewing, smiling, speaking, and psychosocial wellbeing. Complementing this, the concept of oral health-related quality of life (OHRQoL) has gained prominence. OHRQoL is a multidimensional measure encompassing physical comfort, emotional wellbeing, self-esteem, and social functioning. Research has shown that children with ASD often exhibit significantly lower OHRQoL, which may negatively impact their overall development and psychosocial functioning [2].

Despite increasing attention to this topic, substantial challenges remain in implementing effective oral care strategies that are responsive to the unique needs of children with ASD. Understanding the behavioral, sensory, and communicative barriers that they face is crucial in advancing inclusive and equitable dental care.

To address behavioral challenges in the dental setting, the American Academy of Pediatric Dentistry recommends behavior guidance techniques such as the “Tell–Show–Do” method, alongside both verbal and non-verbal communication strategies [3,4]. This approach has shown promise in improving cooperation and reducing caries and plaque-associated gingivitis in children with ASD.

Recent innovations in oral health strategies for children with ASD include the adaptation of dental environments and interventions to accommodate sensory sensitivities and behavioral needs. Parent training programs have demonstrated efficacy in improving oral hygiene behaviors and reducing dental disease.

Aim: The aim of this narrative review is to synthesize current evidence on the oral health challenges, risk factors, and care strategies for children with ASD, with a focus on identifying common clinical problems, behavioral and sensory-related barriers, caregiver roles, systemic access issues, and emerging interdisciplinary and behavioral interventions. By summarizing key findings across diverse studies, this review seeks to highlight gaps in current practice and propose pathways toward more inclusive and effective oral healthcare for children with ASD.

## 2. Materials and Methods

### 2.1. Study Design

This study is a narrative literature review designed to synthesize the current knowledge of oral healthcare in children diagnosed with ASD. Unlike systematic reviews, narrative reviews allow for the broader inclusion of interdisciplinary perspectives and emphasize interpretive synthesis over statistical aggregation. The review was conducted following the general principles of transparency, relevance, and critical comparison, while acknowledging the inherent limitations of non-systematic approaches.

#### 2.1.1. Search Strategy

A comprehensive literature search was conducted across the following electronic databases: PubMed, Web of Science, Scopus, and PsycINFO. In addition, clinical trial registers (e.g., www.ClinicalTrials.gov) and grey literature sources (e.g., Google Scholar, institutional reports) were reviewed to identify ongoing or unpublished studies. The search was carried out between 25 February 2025 and 25 April 2025.

Search terms combined Medical Subject Headings (MeSH) and free-text keywords related to

- Autism spectrum disorder (ASD);
- Oral hygiene, oral care, dental health;
- Children, adolescents;
- Communication tools, dental interventions, quality of life.

Boolean operators (AND, OR) and appropriate filters (age group, language, publication date) were applied.

#### 2.1.2. Eligibility

##### Inclusion Criteria

- Peer-reviewed articles published between 2010 and 2024.
- Studies involving children and adolescents (0–18 years) formally diagnosed with ASD only, without other comorbidities.
- Publications in English.
- Studies reporting on one or more of the following:
  ○ Oral health status or dental disease prevalence;
  ○ Oral hygiene or preventive care practices;
  ○ Oral health-related quality of life (OHRQoL);
  ○ Access to dental care services;
  ○ Communication strategies or dental professional behavior.
- Full-text availability.

##### Exclusion Criteria

- Studies published before 2010.
- Studies involving adults or individuals with unspecified or multiple developmental disabilities.
- Publications in languages other than English.
- Non-peer-reviewed materials: letters, commentaries, editorials, conference abstracts, and theses.
- Studies focused solely on pharmacological or behavioral interventions unrelated to oral health.

#### 2.1.3. Study Selection Process

An initial pool of 301 articles was identified through database searching. After removing duplicates and screening titles and abstracts for relevance, 256 articles were retained for full-text review. Subsequently, 119 articles were excluded based on the defined criteria (Supplementary Materials), resulting in a final selection of 72 articles included in this review.

**Figure 1:**
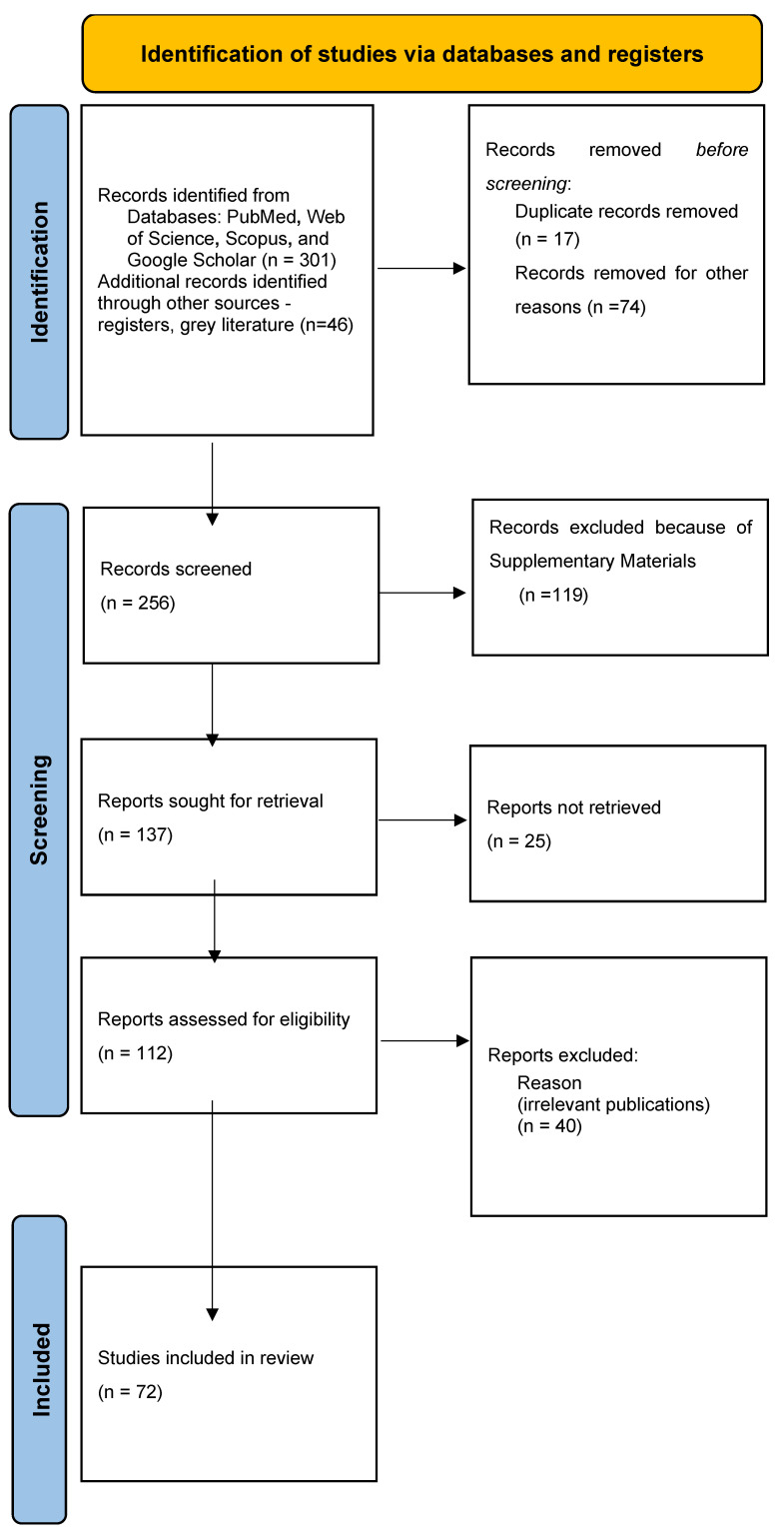
Flowchart of selection.

The article selection process is illustrated in Figure 1.

#### 2.1.4. Data Extraction

During the full-text review process, information from each included study was analyzed and synthesized using a thematic approach. Although a formal data extraction matrix was not preserved, we reviewed and organized the literature based on recurring patterns related to oral health in children with autism spectrum disorder (ASD).

The data were conceptually grouped into the following five major thematic domains, which are reflected in Section 3:

1. Oral health challenges in ASD populations—including the prevalence of dental caries, gingival inflammation, poor oral hygiene, and associated clinical complications such as gingival overgrowth in children with ASD.
2. Behavioral and sensory-related barriers—such as resistance to toothbrushing, sensory sensitivities, communication difficulties, and anxiety related to dental visits, which hinder both home-based oral hygiene and clinical care.
3. Caregiver and family involvement—encompassing parental awareness, the frequency of assisted brushing, challenges in maintaining routines, and the importance of parent-led behavioral management strategies.
4. Dental professional preparedness and communication—such as low rates of prophylactic visits, limited availability of trained professionals, overreliance on emergency interventions, and disparities in healthcare systems across countries and socioeconomic groups.
5. Innovative and interdisciplinary interventions—including the use of sensory-adapted environments, social stories, video modeling, caregiver training, and collaboration with occupational therapists to improve cooperation and oral health outcomes.

This thematic organization allowed for structured, qualitative synthesis and helped to identify both consistent findings across studies and areas where research is still emerging or inconsistent.

## 3. Results

Based on the reviewed scientific literature, a range of consistent characteristics and oral health-related challenges were identified in children diagnosed with ASD.

### 3.1. Oral Health Status and Risk Indicators

The analysis revealed recurring clinical, behavioral, and systemic factors influencing oral health outcomes in this population. The literature reports the high prevalence of dental caries, gingival inflammation, poor oral hygiene, and limited participation in preventive dental care among this population [5,6,7,8,9,10,11,12,13,14,15,16,17,18,19,20,21,22]. Several studies have documented inadequate oral health policies and significant disparities in oral health outcomes compared to neurotypical children [4,5,12]. Research has reported gingival inflammation in more than 50% of children with ASD [3,4]. The underlying causes are thought to include bacterial plaque accumulation, altered immune responses, inflammation, and delays or abnormalities in tooth eruption. Moreover, gingival overgrowth caused by phenytoin—a medication sometimes prescribed for ASD-related seizures—can further impair oral hygiene by physically obstructing access to tooth surfaces [3,4,14,15,16,17,18,19,20,21,22,23,24,25,26,27]. Social stories and visual schedules are commonly used tools that help children to understand the steps involved in dental visits or toothbrushing routines, thereby reducing anxiety and enhancing cooperation [23].

### 3.2. Behavioral and Sensory-Related Barriers

Barriers to adequate oral hygiene among children with ASD include sensory sensitivities, resistance to routine procedures, and communication difficulties [3,5,6,7,8,9,10,11,25,26,27,28,29,30,31,32,33,34]. As a result, daily oral hygiene is often inadequately performed, contributing to increased plaque accumulation, higher gingival index scores, and a greater risk of developing caries and periodontal complications. According to the literature, only 5% of these children undergo annual prophylactic checkups, and 82.7% receive parental assistance for brushing only once daily [6,10,24]. Parental reports often cite difficulties in managing resistant behavior and an inability to influence dental routines, leading to skipped tooth brushing and avoidance of dental care [11]. In addition to clinical findings, the studies discussed the integration of multidisciplinary educational programming as a complementary approach to oral care in children with ASD.

Furthermore, a smaller subset of studies addressed the potential contributions of genetic, environmental, and neurobiological factors in shaping ASD-related oral health vulnerabilities [35,36,37]. Although these studies were not clinical trials, they suggested potential correlations between ASD phenotypes and increased susceptibility to poor oral health outcomes, including atypical oral–motor development, altered salivary compositions, and increased sensory defensiveness. Among the included studies, some reported on the oral hygiene status in children with ASD, using indices such as the Simplified Oral Hygiene Index (OHI-S), Plaque Index (PI), and Gingival Index (GI) [10,38,39]. In three of these studies, children with ASD had significantly higher PI scores (mean difference range: 0.6–1.4; *p* < 0.05) and greater gingival inflammation than neurotypical controls. Three studies using caregiver questionnaires, including the ECOHIS and P-CPQ, indicated that poor oral health impacted daily functioning and caused frequent discomfort.

Five randomized controlled trials evaluated behavioral or educational interventions. For instance, Fenning et al. (2023) reported a 48% increase in the twice-daily brushing frequency and a 35% reduction in plaque scores (*p* < 0.001) in the parent training group versus the psychoeducational toolkit group [40]. Gandhi et al. (2023) found that children who received social story instruction showed significantly improved toothbrushing compliance (*p* = 0.02) and gingival health (GI reduction of 0.4 points) [24,40,41,42,43].

Only three studies included professional perspectives; two of these highlighted gaps in training related to ASD-specific behavioral strategies, and one noted improved practitioner confidence following exposure to visual support tools [44,45,46].

### 3.3. Caregiver and Family Involvement

The reviewed literature also emphasizes the importance of caregiver involvement in daily oral hygiene routines and the implementation of tailored approaches adapted to the sensory sensitivities and behavioral profiles commonly observed in children with ASD [10,14,15,16,17,18,19,20,21,22,24,25,47,48,49,50,51,52,53,54,55,56,57,58,59,60,61,62,63]. These include desensitization protocols, sensory-friendly environments, and appropriate product use (e.g., toothbrush holders, chlorhexidine, fluoride treatments) [14,15,16,17,18,19,20,21,22,24,25]. Parental involvement in daily hygiene routines was emphasized as a determining factor for improved outcomes [10,11,24]. Beyond clinical techniques, educational and behavioral interventions have emerged as essential tools in promoting oral health among autistic populations. Parent training programs have shown significant improvements in brushing frequencies and reductions in the incidence of plaque and caries, especially when caregivers receive structured instruction and reinforcement [40,64].

### 3.4. Dental Professional Preparedness and Communication

Many of the included studies highlight low rates of routine dental visits and limited awareness among parents and caregivers about the relationship between oral and general health. For example, only 5% of children with ASD reportedly receive annual prophylactic checkups, while emergency visits are far more common [6,7,8,9]. Other studies describe limited access to specialized dental services and a high frequency of emergency-based dental interventions [19,20,21,22,32,33,34,65,66,67,68,69,70,71,72,73].

A number of studies underscore the critical need for interprofessional collaboration between dental practitioners, occupational therapists, behavioral specialists, and caregivers, as well as the necessity of enhanced training among dental professionals to effectively address the complex needs of individuals with autism spectrum disorder (ASD) [23,40]

### 3.5. Innovative and Interdisciplinary Interventions

Emerging clinical approaches such as sensory-adapted dental environments, structured desensitization techniques, and individualized oral care plans further complement these behavioral strategies, tailoring the intervention to each child’s sensory profile and developmental level. These multimodal interventions are particularly important given the behavioral and sensory sensitivities associated with ASD, which often interfere with routine dental care.

Similarly, visual pedagogy—such as visual schedules and social stories—has proven effective in reducing anxiety and enhancing cooperation during both home-based and in-office dental procedures [23,41]. Video modeling, where children observe peers or caregivers performing oral care tasks, has also been found to improve skill acquisition and reduce resistance. Behavioral techniques such as “Tell–Show–Do” and positive reinforcement are recommended by the American Academy of Pediatric Dentistry and have been associated with improved outcomes in children with ASD [3,4,74].

Some studies highlighted the involvement of fields such as behavioral psychology, occupational therapy, and digital technologies (e.g., tablet-based visual supports and AI-assisted scheduling tools) in supporting oral hygiene routines and reducing anxiety during dental care. For example, two interventions employed computer-assisted learning modules to enhance the social and procedural understanding related to oral hygiene tasks, resulting in improved cooperation and reduced distress [75,76].

A consistent theme across these studies is the urgent call for more inclusive, accessible, and patient-centered oral health strategies for children with autism spectrum disorder. Several studies highlight the financial burden experienced by the families of children with ASD due to the need for specialized and often inaccessible oral care [25,65,66,67,68,69,70,71,72,73,77,78,79,80].

## 4. Discussion

In parallel to its well-documented effects on general health and development, autism spectrum disorder (ASD) exerts a substantial influence on oral health during childhood. Our review shows that children with ASD frequently exhibit increased rates of dental caries, periodontal disease, and oral trauma, often linked to poor oral hygiene, dietary preferences, self-injurious behaviors, and challenges with cooperation during dental care [2,81]

The concept of health-related quality of life (HRQoL) encompasses both systemic and oral health [82]. Proper general health reflects coordinated function across bodily systems, while optimal oral health involves the effective management of dental caries and its complications, the maintenance of gingival and periodontal health, and the structural and functional integrity of the oral mucosa [4,6,7,8,9,10,11,24,25].

The findings in this review confirm that children with autism spectrum disorder (ASD) experience a disproportionately higher burden of dental disease and impaired oral hygiene compared to their neurotypical peers. This disparity is largely attributed to behavioral challenges, sensory sensitivities, and the inadequate adaptation of conventional dental care models. The absence of proactive, primary preventive measures often results in delayed treatment, necessitating reliance on more invasive and costly secondary and tertiary interventions [2]. Consequently, coordinated, interdisciplinary efforts involving dental professionals, occupational therapists, and caregivers are critical to implement individualized preventive care strategies and reduce the frequency of reactive, crisis-driven treatment.

The lack of primary preventive measures frequently leads to reliance on secondary and tertiary care, which is both more costly and less effective [3]. Therefore, coordinated efforts from dental professionals, occupational therapists, and caregivers are essential to reduce the need for reactive interventions.

These findings support the growing consensus that improving oral health in children with ASD requires more than traditional dental intervention. Multidisciplinary educational approaches—drawing on insights from behavioral science, occupational therapy, and digital learning platforms—offer promising pathways for personalized, child-centered care. Equally, while preliminary, studies investigating the genetic and environmental underpinnings of ASD highlight the need for future research on biological mechanisms linking neurodevelopment and oral health.

Several studies emphasize the importance of integrated educational programming involving multidisciplinary collaboration across fields such as computer science, behavioral therapy, psychology, and occupational therapy. These fields influence daily functioning and personal skills in children with ASD and can be applied to oral care strategies [3]. In parallel, researchers have highlighted the significance of investigating genetic and environmental risk factors influencing ASD manifestations, including possible links with oral health outcomes [47,48,49,50,51,52,53].

There is a growing consensus on the value of primary prevention and long-term oral health planning supported by interdisciplinary collaboration. Such an approach emphasizes desensitization techniques, behavioral guidance, and caregiver training to support oral hygiene habits tailored to the child’s individual needs [54,55,56,57].

Surveys such as the one discussed by Zerman et al. reveal that many children with ASD perceive dental treatment as distressing or traumatic. Distrust in dental interventions and medications further complicates care delivery. This underscores the necessity of prevention-focused strategies, including familiarization with dental settings, regular dental visits, and community-based support to ease both economic and emotional burdens [4,14,15,25,63].

Although the current body of literature on oral health in children with autism spectrum disorder (ASD) lacks strong, large-scale, evidence-based clinical guidelines, emerging research supports several promising strategies informed by interdisciplinary collaboration and practice-based adaptations. Pediatric psychologists, neuropsychiatrists, and physicians play a vital role in facilitating autism-friendly care environments through the development of desensitization protocols, behavior modeling, and sensory integration techniques. While many studies are exploratory or limited by small sample sizes, consistent findings suggest that individualized dental care plans—integrated within broader interdisciplinary frameworks—enhance care accessibility, improve cooperation, and promote better oral health outcomes in this population [23,40].

The clinical expression of ASD symptoms is further influenced by genetic, immunologic, nutritional, and infectious factors. Notably, some researchers have drawn attention to the prevalence of ASD in regions with water fluoridation, including certain areas of Spain and the United Kingdom, prompting further investigation into the potential neurotoxicity of chronic fluoride exposure [26].

Parental attention is often overwhelmed by the complex general healthcare needs of their children, leading to the deprioritization of oral hygiene. Therefore, dentists must play an active role in educating, training, and motivating caregivers to incorporate proper tooth brushing techniques—both manual and electric—into daily routines [65,66,67,68,69,70,71,72,73,74,75,76,77,78,79].

The integration of interdisciplinary collaboration, following a patient-centered model, is essential for the effective prevention and management of oral health conditions in children with ASD. Team-based strategies that include health professionals, educators, therapists, and families offer a sustainable path to improving oral health outcomes and overall quality of life in this vulnerable population [3,65,66,67,68,69,70,71,72,73,74,75,76,77,78,79].

The recommended oral care for autistic children involves several key strategies to address their unique needs and challenges. According to the American Academy of Pediatrics, the following recommendations are essential in promoting oral health in children with developmental disabilities, including autism [64]:

1. Annual Dental Assessments: Dental and periodontal health should be assessed at least annually.
2. Structured Screening: Use structured screening instruments, such as the “oral health risk assessment tool”, to identify risk factors consistently.
3. Anticipatory Guidance: Provide guidance on oral hygiene, diet, habits, trauma prevention, and malocclusion. This includes recommending the use of fluoridated toothpaste, assessing community water fluoridation, applying fluoride varnish as appropriate, and reducing the consumption of fermentable carbohydrates.
4. Dental Home: Advocate for establishing a dental home by 1 year of age, similar to a medical home, and ensure the communication of the child’s intellectual and functional abilities with dental providers.
5. Preventive Dental Care: Encourage families to access preventive dental care regularly.

Additional strategies include the following:

- Caregiver Training: Training caregivers in oral hygiene techniques has been shown to improve outcomes. A randomized controlled trial demonstrated that parent training significantly increased twice-daily toothbrushing and reduced plaque and caries in children with autism [40].
- Adapted Toothbrushes: Using specially adapted toothbrushes, such as those with larger handles or multiple heads, can improve both independent and assisted brushing [1].
- Visual Aids and Social Stories: Employing visual aids, such as social stories, can help to teach toothbrushing skills and improve the oral hygiene status in children with autism [3].
- Sensory Adaptations: Modifying the sensory environment of dental visits, such as using sensory-adapted environments and video modeling, can reduce anxiety and improve cooperation during dental care [74].

These comprehensive strategies, grounded in evidence-based guidelines and clinical trials, are essential in optimizing oral healthcare in autistic children.

## 5. Strengths and Limitations

### 5.1. Strengths

One of the primary strengths of this narrative review is the comprehensive scope of the literature search, which included multiple reputable databases (PubMed, Web of Science, Scopus, and Google Scholar) and a wide range of interdisciplinary keywords. This enabled the identification of diverse sources addressing the complex relationship between autism spectrum disorder and oral health from clinical, behavioral, and social perspectives.

Additionally, this review included literature from multiple geographical regions, which provides a more global perspective on the challenges and practices related to oral care in children with ASD. The structured data extraction process allowed for the systematic thematic categorization of the findings, improving the clarity and synthesis. The inclusion of up-to-date sources published from 2010 to 2024 ensures that the review reflects the most current knowledge and practices in the field.

### 5.2. Limitations

As a narrative review, this study is inherently limited by the lack of formal systematic review protocols, such as PRISMA-compliant search and quality appraisal tools. This may introduce selection bias and reduce the reproducibility. The exclusion of non-English literature might have led to the omission of relevant studies published in other languages. While the narrative design allows flexibility in source selection and thematic synthesis, it inherently limits reproducibility and risk-of-bias assessment. The lack of a standardized appraisal tool and inclusion criteria may introduce selection bias. These limitations were partially mitigated through cross-validation between the authors and the triangulation of evidence from multiple sources.

Another limitation is that the review focused only on children with ASD without comorbidities, which may limit the generalizability of findings to the broader ASD population, where co-occurring conditions are common. Furthermore, many of the included studies were cross-sectional or survey-based, limiting the ability to draw causal inferences or assess longitudinal outcomes.

## 6. Conclusions

Children with autism spectrum disorder face significant challenges in receiving adequate oral hygiene care due to the complex nature of their condition. Due to the complex and multifaceted characteristics of autism spectrum disorder observed in children globally, many of these individuals are deprived of consistent and appropriate oral hygiene care. This places them at a significantly higher risk of developing oral health problems, including dental caries, gingival inflammation, and poor oral hygiene-related quality of life.

There is a clear and urgent need for collaborative efforts between parents, caregivers, dental professionals, and interdisciplinary specialists to address the daily challenges faced in maintaining oral health in children with ASD. A patient-centered, tailored approach—supported by education, early prevention, and integrated care strategies—can substantially improve oral health outcomes and contribute to the overall wellbeing and quality of life of children with autism spectrum disorder.

## Supplementary Materials

The following supporting information can be downloaded at: https://www.mdpi.com/article/10.3390/children12060750/s1, Table S1. Reasons for exclusion after full-text review.

## Abbreviations

AAPD	American Academy of Pediatric Dentistry
ASD	Autism Spectrum Disorder
dft	Decayed and Filled Primary Teeth Index
DMFT	Decayed, Missing, and Filled Permanent Teeth Index
GI	Gingival Index
Ind.	Indicators
Interv.	Intervention
OHRQoL	Oral Health-Related Quality of Life
PI	Plaque Index
Pop.	Population
PRISMA	Preferred Reporting Items for Systematic Reviews and Meta-Analyses
QoL	Quality of Life
WHO	World Health Organization
