# Supplementary material for: Significance of Oral Care for Children with Autism Spectrum Disorder—A Narrative Literature Review
Source: Children (Basel). 2025 Jun 9;12(6):750. doi: 10.3390/children12060750 (PMC12191831; doi:10.3390/children12060750)
Supplement: Supplementary file 1 [file children-12-00750-s001.zip › children-3617714-supplementary.pdf]

Supplementary Materials Table S1. Reasons for Exclusion After Full-Text Review.

**Reason for Exclusion**	**n**
Presence of comorbidities or mixed disabilities	44
Participants older than 18 years or adult populations	18
No specific focus on oral health or dental care	21
Publication type (editorials, letters, commentaries, theses, etc.)	11
Non-English language	6
Pharmacological or behavioral studies with no oral health component	9
Full text not available	10
Duplicates missed in initial cleaning	5
Studies published before 2010	4
Low methodological quality (e.g., insufficient data, unclear outcomes)	9
**Total excluded after full-text review**	**119**
